# Postembryonic Nephrogenesis and Persistence of Six2-Expressing Nephron Progenitor Cells in the Reptilian Kidney

**DOI:** 10.1371/journal.pone.0153422

**Published:** 2016-05-04

**Authors:** Troy Camarata, Alexis Howard, Ruth M. Elsey, Sarah Raza, Alice O’Connor, Brian Beatty, Jack Conrad, Nikos Solounias, Priscilla Chow, Saima Mukta, Aleksandr Vasilyev

**Affiliations:** 1 Department of Biomedical Sciences, NYIT College of Osteopathic Medicine, Old Westbury, New York, United States of America; 2 Department of Anatomy, NYIT College of Osteopathic Medicine, Old Westbury, New York, United States of America; 3 Louisiana Department of Wildlife and Fisheries, Grand Chenier, Louisiana, United States of America; The Roslin Institute, UNITED KINGDOM

## Abstract

New nephron formation (nephrogenesis) ceases in mammals around birth and is completely absent in adults. In contrast, postembryonic nephrogenesis is well documented in the mesonephric kidneys of fishes and amphibians. The transient mesonephros in reptiles (including birds) and mammals is replaced by the metanephros during embryogenesis. Thus, one may speculate that postembryonic nephrogenesis is restricted to the mesonephric kidney. Previous reports have suggested the metanephros of non-avian reptiles (hereafter reptiles) may continually form nephrons throughout life. We investigated the presence of adult nephrogenesis in reptiles by examining adult kidneys from several species including *Trachemys scripta*, *Chrysemys picta*, *Boa constrictor*, *Tupinambis tegu*, *Anolis carolinensis*, and *Alligator mississipiensis* among others. We found that all major reptilian groups (Testudines, Crocodylia, and Squamates) showed the presence of adult nephrogenesis. The total amount of nephrogenesis varied greatly between species with turtles displaying the highest density of nephrogenesis. In contrast, we were unable to detect adult nephrogenesis in monotremes, and in the iguanid *A*. *carolinensis*. Nephron progenitor cells express the transcription factor Six2, which in mammals, becomes downregulated as the progenitor cell population is exhausted and nephrogenesis ends. Using the alligator as a model, we were able to detect Six2-positive cap mesenchyme cells in the adult kidney, which spatially correlated with areas of nephrogenesis. These results suggest that the metanephric kidney of reptiles has maintained the ability to continually grow new nephrons during postembryonic life, a process lost early in mammalian evolution, likely due to the persistence of a Six2-expressing progenitor cell population.

## Introduction

The vertebrate kidney has evolved to regulate water homeostasis and waste excretion to maintain our *milieu interieur* [1]. Three types of kidney structures have developed over the course of vertebrate evolution, the pronephros, mesonephros, and metanephros. In fish and amphibians, the paired epithelial tubules of the embryonic pronephros are followed by the development of the more complex mesonephric kidney found in the adult. In amniotes, the pronephros and mesonephros become reabsorbed, and are replaced by the final metanephric kidney. The overall structural complexity of each kidney type increases from a parallel pair of nephrons in the zebrafish pronephros [2] up to a million highly organized nephrons in a human metanephric kidney [3]. However, the segmentation pattern of each kidney type appears conserved as the nephrons of the pro-, meso-, and metanephric kidney each have a proximal, intermediate, and distal segment which drain into a collecting duct system with conserved segmental gene expression [4–7].

Cells destined for a renal fate are derived from the intermediate mesoderm (IM) during embryogenesis. For example, during morphogenesis of the zebrafish pronephros, cells from the IM go through a mesenchymal-to-epithelial transition (MET) to form the pronephric duct epithelium and tubule formation progresses in an anterior-to-posterior axis [2]. The pronephric tubule is then patterned with a segmented structure highly reminiscent of the mammalian nephron [4]. Once the pronephric kidney is established and functional, the zebrafish mesonephros forms along the pronephric tubule, again, progressing from anterior to posterior [8,9]. Progenitor cells adjacent to the pronephric tubule condense and elongate to form the mesonephric tubules, which eventually fuse with the pronephric tubule [7]. This process is repeated making a branched mesonephros, where nephrons are sequentially attached to a single mesonephric duct, inherited from the pronephros.

In mammals, the pronephros and mesonephros develop along the nephric duct, progressing from anterior to posterior [10]. The pronephric and mesonephric kidneys degenerate, with cells from the mesonephros contributing to the male gonads. Along the nephric duct, posterior to the mesonephros, the final metanephric kidney begins to form by embryonic day 35 in humans (embryonic day 10.5 in mouse). Development of the metanephric kidney starts when the ureteric bud (UB) branches off the mesonephric duct and invades an overlying metanephric mesenchyme (MM) [11]. Reciprocal signaling between cells of the MM and UB induce nephron morphogenesis. The UB branches with MM condensing at each branch tip and undergoing MET to produce renal vesicles which elongate and connect to the UB [12,13], and mature into nephrons. The MM gives rise to the glomerular epithelium, proximal tubule, Loop-of-Henle, and distal tubule segments, while the UB branches forming the collecting duct system. This process of UB branching and MM condensation and differentiation continues until the pool of MM progenitor cells becomes exhausted around week 35 of gestation in humans [14] or post-natal day 3 in mice [15].

Consequently, mammals are born with a finite number of nephrons and are incapable of generating new tubules for either tissue homeostasis or repair from injury. The halt in nephron endowment appears to be universal in mammals, as even in examined marsupial species (*Dasyurus hallucatus* and *Trichosurus vulpecula*) [16,17] nephrogenesis stops prior to weaning. The subsequent increase in total functional capacity of the mammalian kidney occurs through nephron hypertrophy and hyperplasia rather than through increase in nephron number (nephrogenesis; [18]).

This developmental switch from nephrogenesis to hypertrophy limits the kidney’s ability to regenerate after acute and chronic injury and the active nephron number declines throughout life due to chronic injury and nephron scarring [19,20]. In humans, if the cumulative injury is extensive enough, this may lead to chronic renal failure [21]. Currently, the only solution for these patients is renal transplantation or dialysis, both of which present significant problems [22]. Thus, it becomes important to understand the ‘lost art’ of continuous nephrogenesis. This knowledge may allow us to develop novel regenerative approaches to reverse nephron loss and enable organ engineering solutions for recovering lost kidney function [23].

In contrast to mammals, the adult mesonephric kidney of osteichthyes, chondrichthyes and lissamphibians is capable of continual nephrogenesis [24]. Several species have been found to add new nephrons to their adult mesonephric kidney, including goldfish [25], catfish, trout, tilapia, toadfish [26], zebrafish [8,9], medaka [27], frogs (*Rana Temporaria*; [28], dogfish [29,30], and skates [31]. Furthermore, the process of nephrogenesis in adult mesonephric kidneys is enhanced following injury [8,9,27,32,33] providing an excellent mechanism for kidney repair. It appears that the continual addition of nephrons to the kidney is a common feature in vertebrates with a mesonephric kidney. Therefore, it was proposed that continual nephrogenesis is specific to mesonephric kidney and is lost in the metanephric kidney of amniotes [34].

However, evidence in the literature suggests that in isolated species of reptiles and birds, the metanephric kidney is capable of nephrogenesis in the post-embryonic period [35–38]. We set out to determine the extent and the prevalence of post-embryonic and adult nephrogenesis in reptiles, aiming to unambiguously establish whether the phenomenon of adult nephrogenesis was restricted to the mesonephric kidney, or if it was also commonly present in the metanephric kidney of reptiles.

We collected kidneys from juvenile and adult reptiles from all of the major reptilian groups including Archosauromorpha (here represented by the non-avian archosaur clade Crocodylia), Testudines and Squamata. We identified histological evidence of juvenile and adult nephrogenesis in all of these groups. However, adult nephrogenesis appears to have been lost in some reptile species, similar to mammals. In addition, we found immunofluorescence evidence of persistent Six2-expressing cap mesenchyme cells. Six2 expression is normally lost in mammals once nephrogenesis ceases, suggesting nephron progenitor cells are present in the reptilian kidney post-embryonically. We conclude that the metanephric kidney is capable of continual nephrogenesis in many adult species of reptiles and this ability was lost very early in, or prior to mammalian evolution. Unfortunately, few “living fossils” of early mammals exist, leaving the question of when this loss occurred in early mammals unanswerable at any node prior to the diversification of monotremes. Thus, we sampled the available specimens of monotremes, but focused our efforts on nonmammalian amniotes.

## Results

### Reptiles maintain nephrogenesis throughout life

Previous reports have suggested the occurrence of new nephron growth, termed nephrogenesis, in reptiles post-embryonically [35–37]. Two of these studies indirectly assessed new nephron formation by estimating glomerular number as a function of animal age. However, Solomon [36] did show histological evidence of nephrogenesis in an adult green sea turtle (*Chelonia mydas*). We questioned whether nephron formation in juvenile or adult reptiles was species specific or a broader phenomenon.

**Fig 1:**
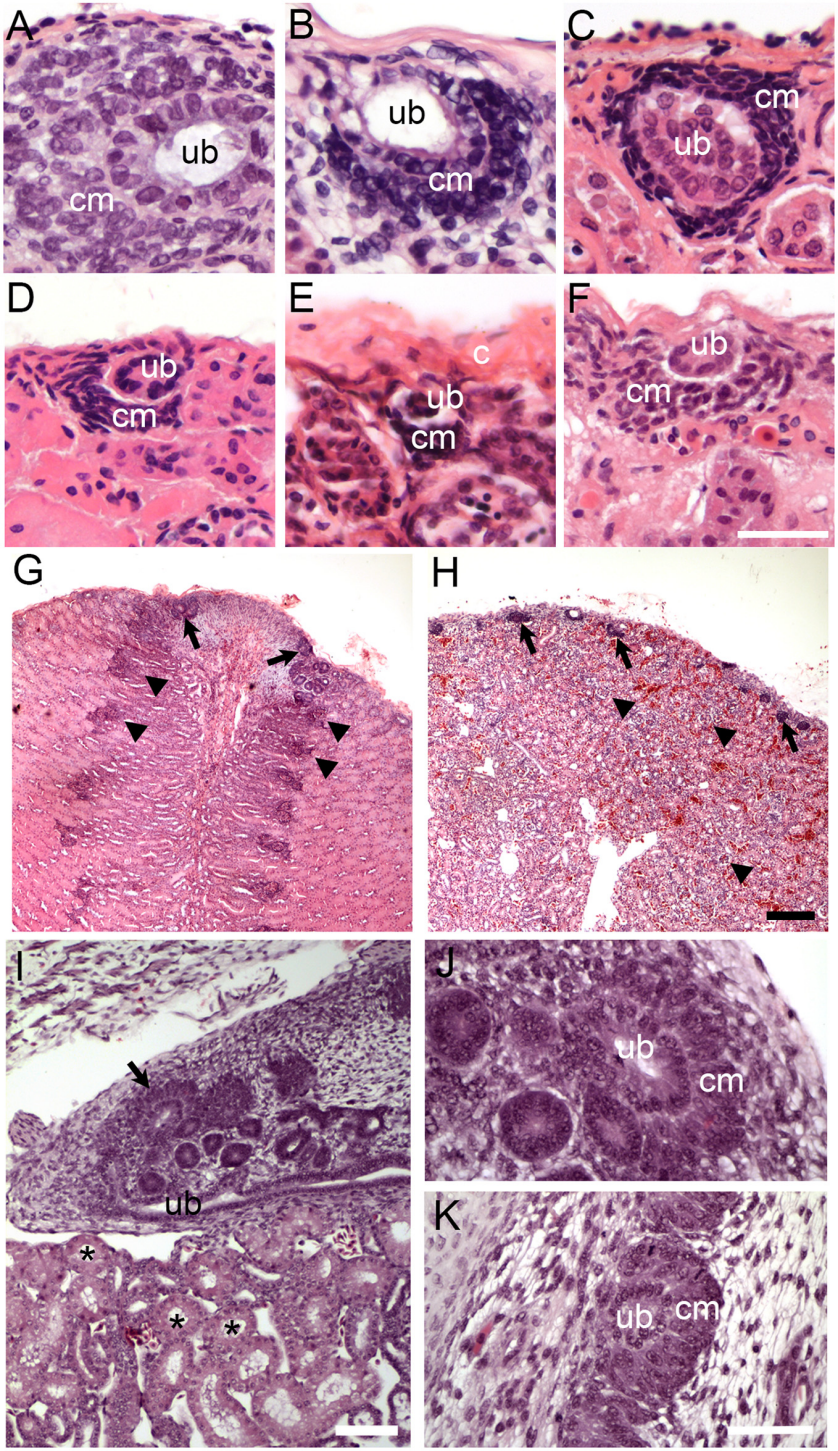
Examples of reptilian post-embryonic nephrogenesis. (A-H) Kidney tissue sections stained with H&E showing zones of nephrogenesis. (A-F) High magnification of zones of nephrogenesis from juvenile American alligator (*A*. *mississippiensis*, A), adult turtles: red-eared slider (*T*. *scripta*, B) and painted turtle (*C*. *picta*, C), adult tegu (*T*. *teguixin*, D), adult Egyptian Mastigure (*U*. *aegyptia*, E), and adult boa constrictor (*B*. *constrictor*, F). Corresponding lower magnification images are shown in S1 Fig. Scale bar = 50 μm. (G-H) Low magnification images showing tissue organization and variability in arrangement of zones of nephrogenesis between American alligator (G) and red-eared slider (H). Arrows denote zones of nephrogenesis, arrowheads point to mature glomeruli. Scale bar = 100 μm. (I-K) Embryonic alligator kidney (stage 18–19). (I) Wide-field view of embryonic alligator mesonephros (bottom; asterisks label mesonephric tubules) and metanephros (top). Zone of nephrogenesis is indicated by the arrow. Scale bar = 100 μm. (J-K) High magnification of nephrogenesis in embryonic alligator metanephric kidney. Scale bar = 20 μm. Ub = tip of the ureteric bud branch, cm = metanephric cap mesenchyme, c = capsule.

Therefore, we obtained adult and juvenile kidney tissue from species found in the major reptilian groups Crocodylia (alligators and crocodiles), Testidines (turtles), and Squamates (lizards and snakes, S1 Table). Histological analysis of tissue sections revealed post-embryonic nephrogenesis in a number of juvenile and adult reptiles, including American alligator (*A*. *mississippiensis*), red-eared slider (*T*. *scripta*), painted turtle (*C*. *picta*), African spiny-tailed lizard (*C*. *tropidosternum*), tegu lizard (*T*. *teguxin*), rock monitor (*V*. *albigularis*), Egyptian mastigure (*U*. *aegyptia*), Chinese water dragon (*P*. *cocincinus*), boa constrictor (*B*. *constrictor*) and western rat snake (*P*. *obsoletus*) (S1 Table). Nephrogenesis was detected as areas of condensed mesenchyme surrounding a tip of terminal duct branch just under the renal capsule (Fig 1*A*–1*H*, also S1 Fig). There was some variability in the size and the location of cap mesenchyme (especially in the case of *U*.*aegyptia*, Fig 1*E*). At least some of the variability could be attributed to the freezing and drying artifact (specimens in Fig 1C–1H have been stored frozen several weeks before processing). However, some of the variability may be reflective of the differences in the overall activity of these nephrogenic zones. It should be also noted that cap mesenchyme aggregates were often seen on the side of the ureteric bud or on the side opposite the capsule with respect to the ureteric bud (contrasting it to the mammalian embryonic development, where cap mesenchyme is most commonly positioned between the ureteric bud and the capsule). This can be only partially explained by random sectioning. Another partial reason is the difference in overall geometry or the reptilian kidney, where ureter is often positioned in the subcapsular location, extending parallel instead to perpendicular to the surface of the kidney. However, there might be other explanations for the observed differences.

**Fig 2:**
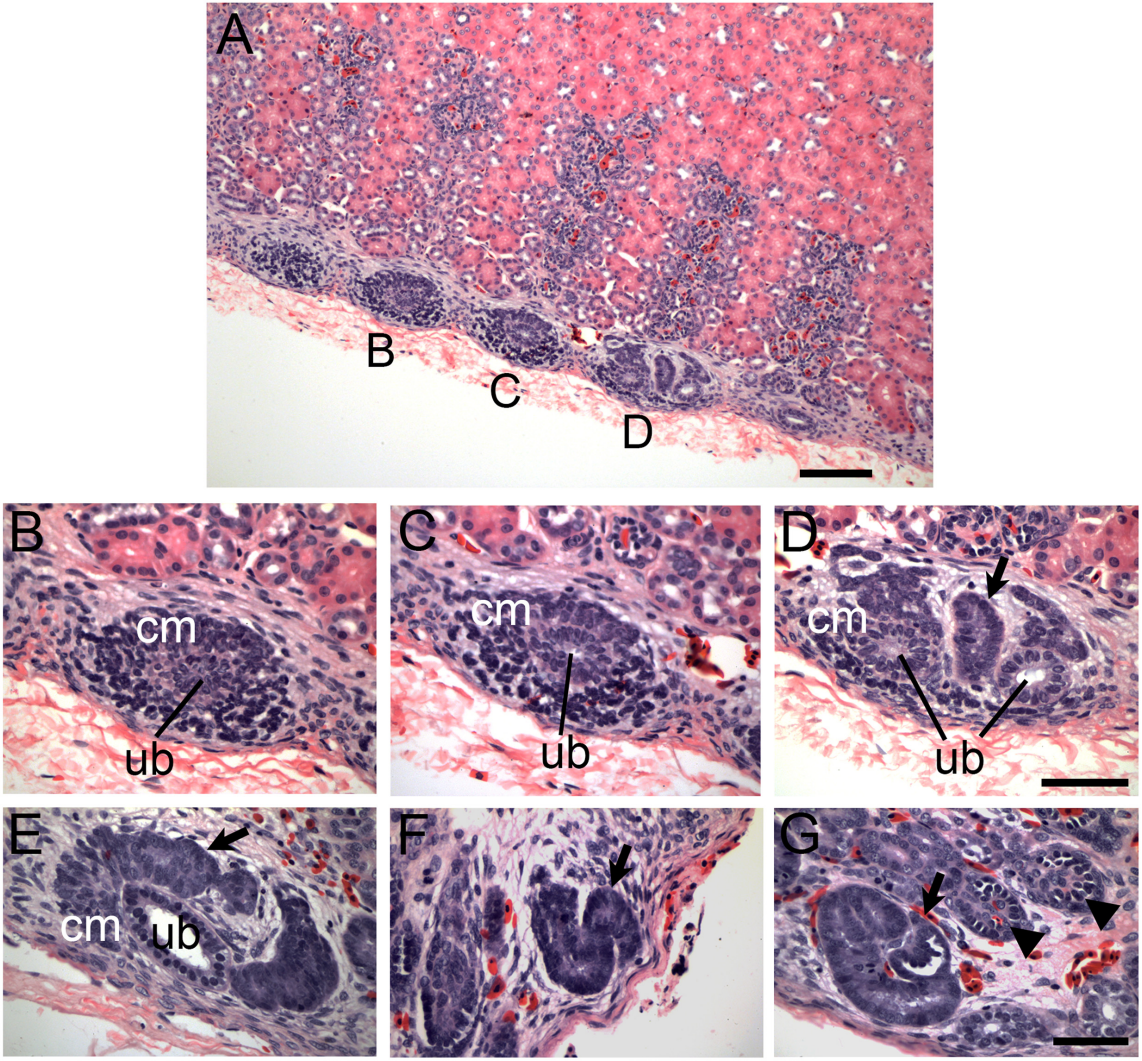
Nephron formation in adult reptiles resembles embryonic nephrogenesis. (A-G) Adult alligator kidney tissue sections stained with H&E. (A) Wide field view of zones of nephrogenesis on the periphery of the kidney just under the capsule. Scale bar = 100 μm. (B-D) Higher magnification of nephrogenic events as denoted in (A). Early stage nephron formation in (B) which progresses to developed condensed mesenchyme in (C) and a later stage nephrogenesis event in (D) with condensing mesenchyme, ureteric bud-like branch tips (ub) and a newly formed immature nephron (arrow). (E-G) Nephrogenesis in adult alligator is reminiscent of embryonic nephron formation. (E) Metanephric cap mesenchyme undergoing MET to form early nephron structure (arrow). (F) S-shaped developing nephron (arrow) similar to S-shaped bodies detected during embryonic nephron formation. (G). Maturing glomeruli. Newly formed glomerulus (arrow) at the end of a newly formed tubule with progressively more mature glomeruli (arrowheads). Scale bar = 50 μm. Ub = tip of the ureteric bud branch, cm = metanephric cap mesenchyme. The lower magnification images corresponding to (E-G) are shown in S2 Fig.

**Fig 3:**
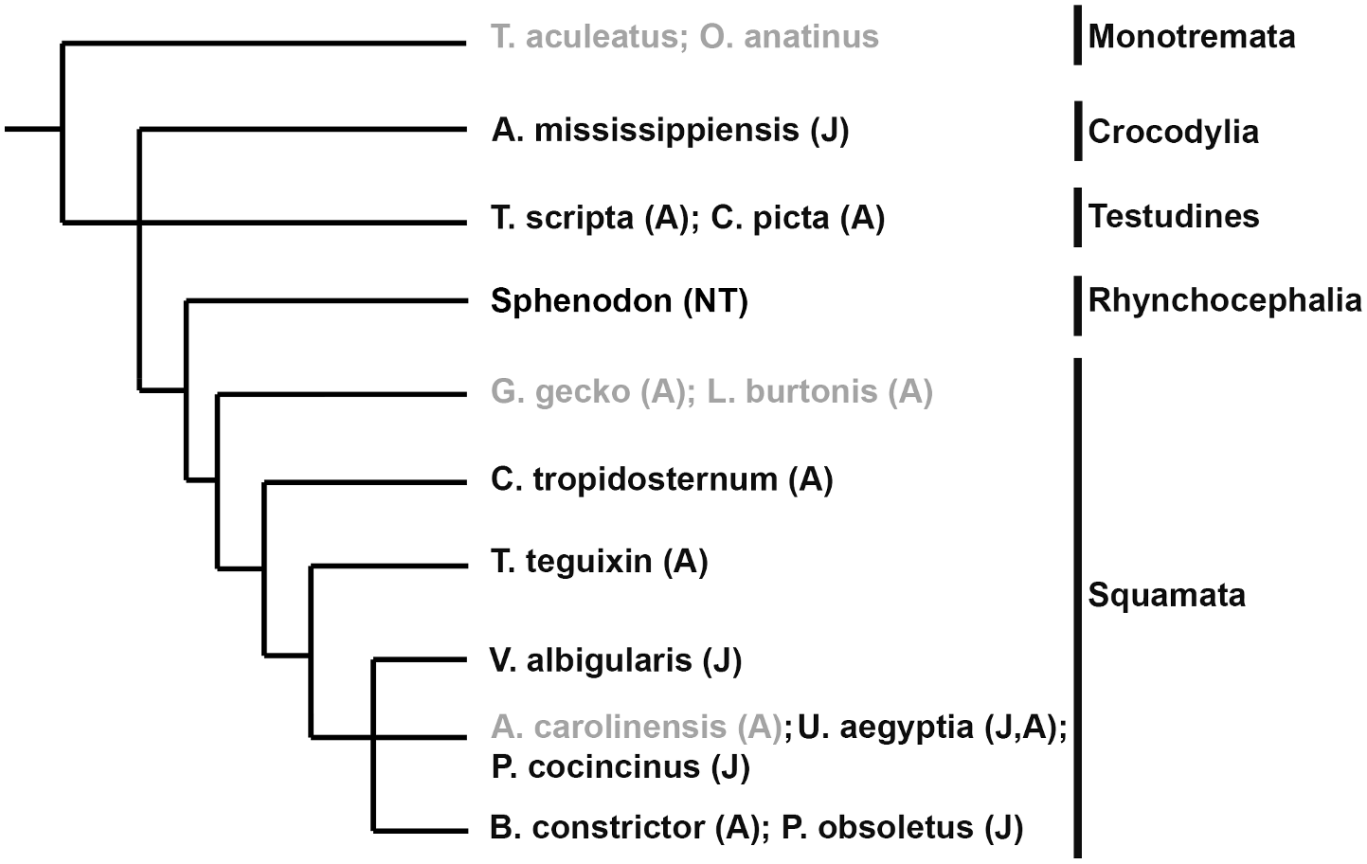
Species surveyed for post-embryonic nephrogenesis. Cladogram displaying species tested for presence of nephrogenesis in juvenile (J) or adult (A) kidney. Two species of monotremes, *Tachyglossus aculeatus* (short-beaked echidna) and *Ornithorhynchus anatinus* (platypus), were used for comparison as the most basal mammalian group. As with other mammals, no evidence of nephrogenesis was detected in the monotreme species. Post-embryonic nephrogenesis was detected in all major reptilian groups surveyed. Species names in light gray did not display evidence of nephrogenesis. NT = not tested.

The overall histology of zones of nephrogenesis detected in juvenile and adult reptiles was similar to that observed during embryonic development (Fig 1I–1K). For example, in juvenile and adult alligators, condensed mesenchymal cells were detected surrounding tips of terminal duct branches (Fig 1A and 1G; Fig 2). This histology recapitulated embryonic nephrogenesis seen in mammals and reptiles, as demonstrated in Fig 1*I*–1*K*. The progression of nephrogenesis in an adult alligator specimen was similar to embryonic nephron formation (Fig 2). Multiple zones of nephrogenesis were found under the renal capsule along the periphery of the kidney (Fig 2*A*). Nephrogenesis appeared to be asynchronous as we detected different stages of nephron formation in adjacent zones (Fig 2*B*–2*D*). Typical sequential forms of nephron development could be detected, including renal vesicle, S-shaped body and various stages of glomerular maturation (Fig 2*E*–2*G*, also S2 Fig). Thus, we were able to detect histological evidence of postembryonic and adult nephrogenesis in a wide range of reptilian species, representing all major reptilian groups (Crocodylia, Testudines and Squamata, summarized in Fig 3).

For comparison, we sampled kidneys from two adult species of monotremes, the short-beaked echidna (*Tachyglossus* aculeatus) and the platypus (*Ornithorhynchus anatinus*) (Fig 3; S3 Fig). Similar to other species of mammals, we did not find evidence of nephron formation in this basal mammalian group. This suggests that the ability to continually grow nephrons after birth was lost very early in the evolution of mammals, although more extensive sampling would be required to establish the timeline of the disappearance of nephrogenesis in monotremes as a function of age.

### Postembryonic nephrogenesis is not universal among reptiles

**Fig 4:**
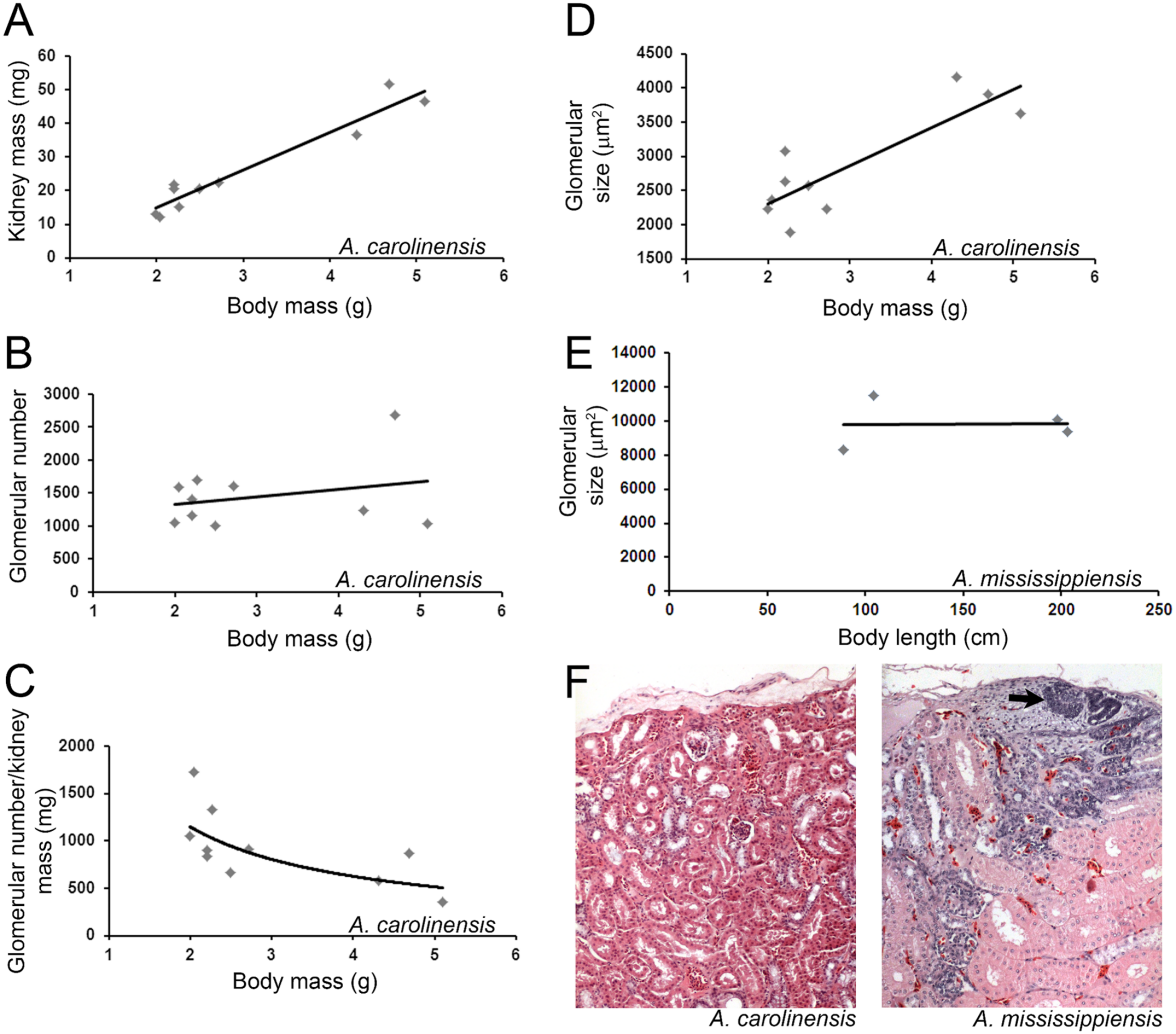
Kidney growth occurs by hypertrophy or nephrogenesis in reptiles. (A-C) Green anole (*A*. *carolinesis*) kidney measurements compared to body mass. (A) Scatter plot of kidney mass related to body mass with line of best fit. (B) Scatter plot of estimated glomerular number related to body mass. (C) Scatter plot of the ratio of glomerular number/kidney mass compared to body mass. Number of biological samples = 10. (D-E) Scatter plots of glomerular size compared to body mass in green anole (D) or body length in American alligator (*A*. *mississippiensis*) (E). (F) Histology of adult green anole kidney (left) and adult American alligator kidney (right). Arrow denotes zone of nephrogenesis in the American alligator. (g) = grams. (cm) = centimeters.

In addition to monotremes, we could not detect evidence of nephrogenesis in gecko (*Gekko gecko*), legless lizard (*Lialis burtonis*), or green anole (*A*. *carolinesis*), suggesting this ability may have been lost in these species or was undetectable under normal physiological conditions (Fig 3, grey text, S4 Fig). The examined specimens of gekkota were limited in number and histological quality making it difficult to draw conclusions. In contrast, when we indirectly assessed the presence of adult nephrogenesis by estimating the total glomerular number in the green anole (*A*. *carolinesis*), the results indicated that nephron number did not change with increased size of the animal (Fig 4). In green anoles, kidney mass was linearly proportional to body mass (Fig 4*A*). However, the estimated number of glomeruli remained constant as body mass increased (Fig 4*B*). Furthermore, the ratio of glomerular number to kidney mass decreased as animals became larger (Fig 4*C*). This was highly suggestive that the adult green anole does not increase kidney size by nephrogenesis. Post-embryonic kidney growth in mammals is accomplished by nephron hypertrophy. To gain insight into whether the green anole uses a similar mechanism to increase renal size, we compared estimated glomerular size to body mass (Fig 4*D*). As expected for nephron hypertrophy, glomerular size increased in relation to body mass.

These data, along with the lack of new nephron endowment, suggest that the green anole, similar to mammals, uses nephron hypertrophy rather than nephrogenesis, to increase kidney size during adult growth. In contrast, when we estimated glomerular size in juvenile and adult American alligator (Fig 4*E*), which have robust nephrogenesis (Figs 1 and 2), we did not detect an increase in glomerular size with increasing body length (up to 2.03 m, Fig 4*E*).

It is interesting to note that the green anole did not show evidence of adult nephrogenesis despite related species within the same suborder, which did display adult nephrogenesis (Fig 3). We detected nephrogenesis in adult Egyptian mastigure (Figs 1 and 3), and nephrogenesis has been proposed to exist in the adult green iguana (*I*. *iguana*; [35] and Yarrow’s spiny tail lizard (*Sceloporus jarrovii*; [37]. Further examination is needed to determine if the green anole completely lacks adult nephrogenesis, similar to mammals, or if continual nephron endowment can be induced by nephron loss or kidney injury.

### Frequency of nephrogenesis varies between reptilian groups

**Fig 5:**
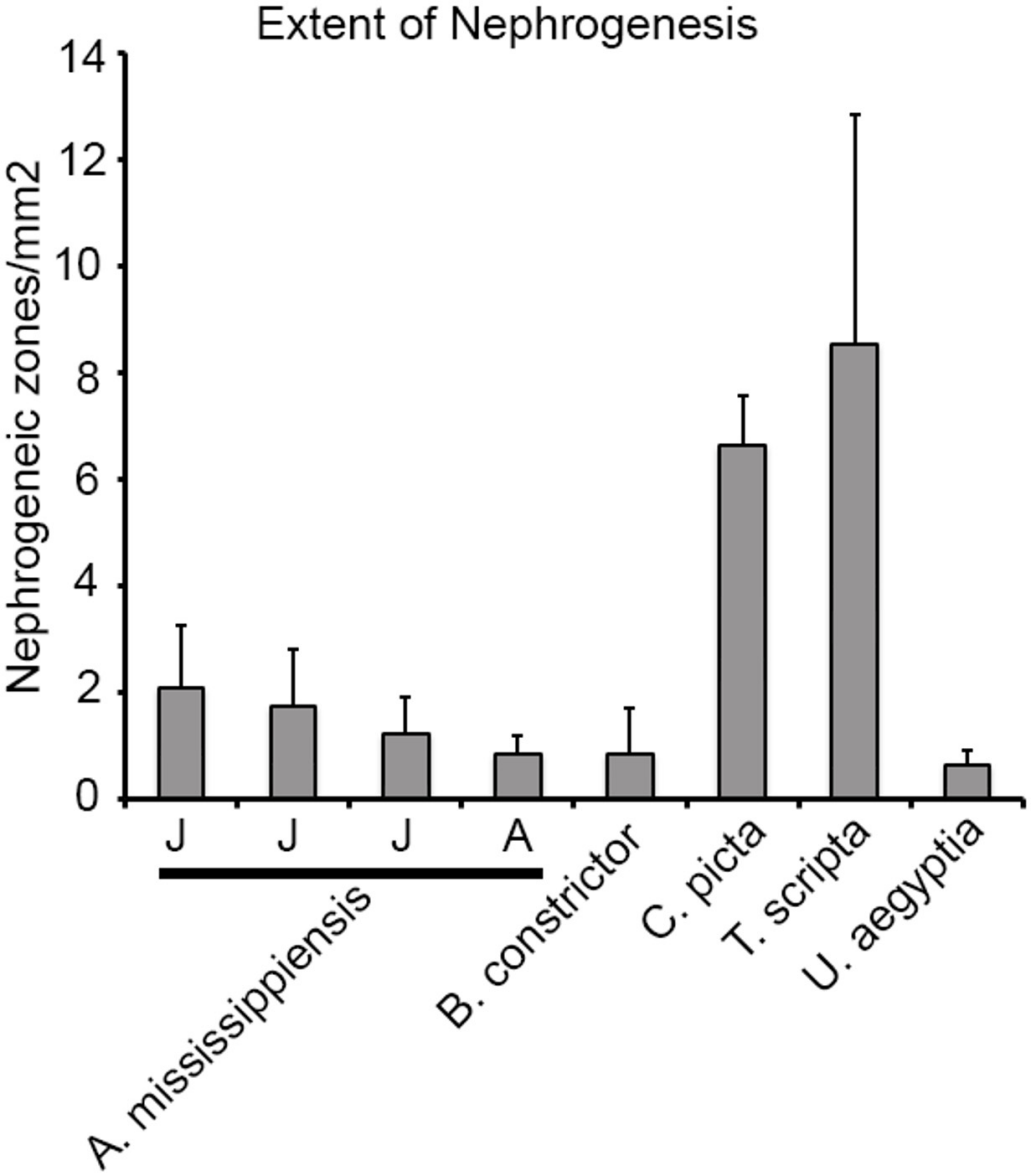
Extent of nephrogenesis in reptiles. The number of nephrogenic zones in each species was normalized to the effective surface area of each section analyzed (methods). The ratio of the total number of nephrogenic zones and the effective surface area was then used to compare the extent of post-embryonic nephrogenesis between different reptile species. Only the material with sufficient preservation to accurately estimate the number of nephrogenesis events was used for analysis. Species analyzed were: *A*. *mississippiensis* (3 juvenile (J), 1 adult (A)), *B*. *constrictor*, *C*. *picta*, *T*. *scripta*, *U*. *aegyptia*. The corresponding density of nephrogenesis: (*A*. *mississippiensis*: 2.1 ± 1.1 (0.89m juvenile), 1.8 ± 1.1 (1.04m juvenile), 1.3 ± 0.7(1.63m juvenile), 0.86 ± 0.35(1.98m adult). *B*. *constrictor*: 0.9 ± 0.9. *C*. *picta*: 6.7 ± 0.9. *T*. *scripta*: 8.6 ± 4.3. *U*. *aegyptia*: 0.7 ± 0.3).

In our study, the majority of juvenile and adult reptilian species displayed evidence of nephrogenesis. However, the gross morphology and extent of new nephron formation varied. We counted zones of nephrogenesis in the specimens where adequate and reliable counts could be obtained, and compared them between species by normalizing to the effective surface area (Fig 5; see Methods). The two adult turtle species, *C*. *picta* and *T*. *scripta*, had the greatest average number of nephrogenic zones per unit surface area (6.7 ± 0.9 /mm^2^ and 8.6 ± 4.3 /mm^2^ respectively, Fig 4). The juvenile alligator kidneys averaged between 1.3 and 2.1 /mm^2^, adult alligator (1.98 m), 0.86 ± 0.35 /mm^2^ followed by the boa (*B*. *constrictor*), 0.9 ± 0.9 /mm^2^ and the Egyptian mastigure (*Uromastyx aegyptia*), 0.7 ± 0.3 /mm^2^. Thus, there was significant variation in the frequency of nephrogenesis between major reptilian groups. In addition, we found differences in the distribution of nephrogenesis events. Most species surveyed showed random distribution of nephrogenesis with respect to the surface of the kidney. In contrast, the kidney of the American alligator was highly organized [39] with rows of glomeruli leading to the apex of each renal lobe (Fig 1*H*, arrowheads). We observed nephrogenesis events only at the points where rows of glomeruli met the capsule (Fig 1*G*, arrows), forming a distinct line of nephrogenesis that could be detected grossly (S5 Fig). In comparison, in turtles, the kidney had glomeruli distributed throughout the cortex (Fig 1*H*, arrowheads), and the nephrogenesis events were distributed randomly under the capsule (Fig 1*H*, arrows).

### Persistence of Six2-expressing kidney progenitor cell population

The metanephric mesenchyme (MM) in mammals expresses specific genes that mark and maintain the pool of kidney progenitor cells. One such gene is the transcription factor Six2, which labels the nephron progenitor population in the MM and is required for its maintenance [40,41]. If the areas of condensed mesenchyme identified in juvenile and adult reptiles are sites of continual nephron endowment, then those cells should express progenitor cell MM markers such as Six2.

**Fig 6:**
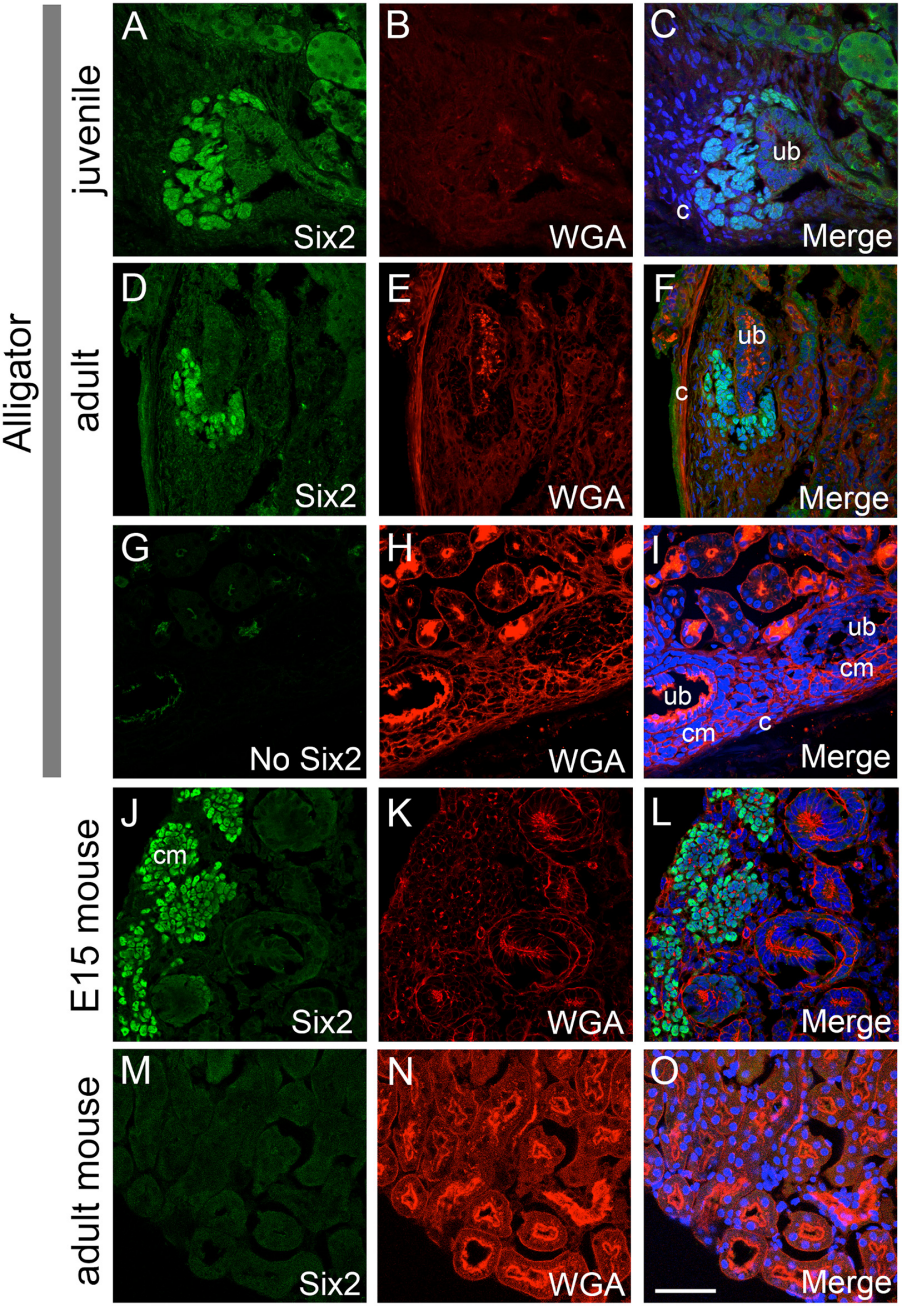
Nephrogenic zones express kidney progenitor marker Six2. (A-I) Confocal immunofluorescence imaging of American alligator sections stained with Six2 antibodies. (A-C) Juvenile alligator kidney stained with Six2 (A) and wheat germ agglutinin (WGA) Alexa Fluor 555, used as a non-specific fluorescent counter-stain (B), merged image with DAPI in (C). (D-F) Adult alligator kidney stained with Six2 (D), WGA555 (E), and merged image with DAPI (F). (G-I) Juvenile alligator kidney stained with Alexa Fluor 488 secondary antibody alone (F), WGA555 (H), and merged image with DAPI in (I). (J-L) Kidney from mouse embryonic day 15 (E15) were similarly stained with Six2 antibodies (J) and WGA555 (K). Image merged with DAPI stain is shown in L. (M-O) Adult mouse kidney stained with Six2 antibodies (M) and WGA555 (N) using the same protocol. Image merged with DAPI stain is shown in O. The apparent cell clustering in (A,C, J and L) is due to freeze artifact. Ub = tip of the ureteric bud branch, cm = metanephric cap mesenchyme, c = capsule. Scale bar = 50 μm.

We stained sections from juvenile and adult alligator kidneys with a polyclonal antibody directed against human Six2 protein, which is over 90% identical in amino acid sequence to alligator Six2 (S6 Fig). On sections from juvenile and adult alligator kidneys, Six2 was specifically localized to the condensed mesenchyme cells detected by histology (Fig 6*A*–6*F*). The Six2 positive mesenchymal cells were adjacent to a terminal ureteric bud branch at the location where rows of glomeruli met the capsule (Fig 6*C and* 6*F*). *Six2* expression was also confirmed by RT-PCR from adult alligator kidney tissue (S6 Fig). The negative control sections stained without primary antibody (Six2) showed no significant fluorescence (Fig 6*G*–6*I*). The localization of the Six2 expressing mesenchyme in alligator sections correlated with zones of nephrogenesis detected by histology, and was very reminiscent of embryonic nephrogenesis in mammals. For comparison, we stained embryonic day 15 (E15) mouse kidneys with Six2 antibodies (Fig 6*J*–6*L*). As expected, Six2 expression was localized to the cap mesenchyme cell population at the periphery of the developing mouse kidney. In contrast, no Six2 was expressed in kidneys from adult mice (Fig 6*M*–6*O*). We also did not observe Six2 antibody staining in adult *A*. *carolinensis* kidneys (S7 Fig), although this result may need additional confirmation by making comparison to a stage in anolis development (juvenile or embryonic) during which nephrogenesis can still be observed. Based on these findings, reptiles appear to maintain continual nephrogenesis throughout life, and this ability correlates with the presence of a progenitor cell population, as evidenced by the expression of Six2 transcription factor.

## Discussion

### Continual nephron formation in vertebrates

It has been thought that the metanephric kidney of amniotes forms a finite number of nephrons just before or after birth. Here, we provide the first systematic study of continual nephrogenesis in the metanephric kidney of reptiles. Nephrogenesis was detected at the periphery of juvenile and adult kidneys in most of the reptile species surveyed (Figs 1–3; S1 and S2 Figs). This is in sharp contrast to mammals, where nephrogenesis terminates at a specific point in development, for example, week 35 of gestation in humans [14], postnatal day 3 in mouse [15], and just prior to weaning in marsupials [16,17]. Similar to studied mammals, we did not detect evidence for continual nephron formation in two species of monotremes, even though the monotremes are likely the mammalian group most closely positioned to reptiles due to their intermediate phenotype, including the kidney [42].

Continual nephron formation has been detected in a number of vertebrates with a mesonephric kidney, such as teleosts [8,9,25–27], amphibians [28], and elasmobrachii [31,33]. Progenitor cells competent for nephrogenesis are maintained in these species throughout adulthood to continually add nephrons and increase organ size. The process of continual nephron formation in the mesonephros also becomes enhanced following an episode of injury. Acute kidney injury induced by gentamicin injection has been shown to increase nephrogenesis in goldfish [32], medaka [27], and zebrafish [8,9]. Partial nephrectomy in the little skate also enhances new nephron formation (33). It appears that continual nephrogenesis is a common theme in the mesonephric kidney as a mechanism to increase nephron number and recovery from injury.

Indirect evidence had suggested that nephrogenesis in adult vertebrates was not limited to the mesonephric kidney. Continual nephron formation is thought to occur in the green iguana [35] and Yarrow’s spiny lizard [37] based on increased estimated glomerular number with increased body mass. However, the only direct demonstration of the presence of nephrogenic zones came from the adult green sea turtle where areas of condensed mesenchyme were detected at the periphery of the kidney [36].

We have examined presence of juvenile and adult nephrogenesis in a number of reptilian species. Zones of nephrogenesis were detected in kidneys from species representing all major reptilian groups: Crocodylia, Testudines, and Squamata (Figs 1–3; S1 and S2 Figs). Birds, which are archosaurian reptiles closely related to crocodylians, may also possess a metanephric kidney capable of nephrogenesis post-embryonically. In the chicken (*Gallus gallus*), glomerular number continues to increase for the first 12 weeks after birth [38] and the juvenile avian kidney also appears to possess nephrogenic zones similar to what is detected in other reptiles (our unpublished observations). Continual nephron formation in vertebrates therefore appears to be the rule rather than the exception with examples of nephrogenesis found in fish, amphibians, and reptiles (including birds). For currently unknown reasons, mammals appear to have lost this ability very early in their evolution, as evidenced in the examined monotreme specimens where we did not detect evidence of continual nephrogenesis (although a more comprehensive study would be required to determine the time course of the disappearance of nephrogenesis in monotremes).

Evolutionary timing of the loss of nephrogenesis is difficult to constrain because of the lack of extant intermediates between the basal amniote and basal mammals. Synapsida (all vertebrates more closely related to mammals than to reptiles) diverged from Sauropsida more than 311 million years ago [43], but the monotreme-lineage did not diverge from other mammals for another 80 million years [44].

### Nephrogenesis vs. hypertrophy

It has been suggested that continual nephrogenesis, especially in fish, is a mechanism for the kidney to increase filtration capacity in order to keep up with increasing body mass [24]. Mammals, on the other hand, respond to increasing body mass by increasing glomerular filtration pressure and increasing kidney mass by nephron hypertrophy [18]. Thus, continual nephrogenesis may be a mechanism to compensate for low blood pressure, such as in fish, while hypertrophy is an adaptation for species with high blood pressure such as in mammals [24]. We have identified both mechanisms in different species of reptile. The green anole does not demonstrate adult nephrogenesis (we were unable to detect histological evidence of nephrogenesis or increased glomerular number with increased body size) but instead utilizes hypertrophy. Glomerular number remained relatively constant in relation to body mass in the green anole, while glomerular size increased (Fig 4). In the American alligator, however, nephrogenesis was robust even in adult animals (Figs 1 and 2) and glomerular size did not increase in relation to body length (Fig 4*E*). Most reptile species have low blood pressure, although some species such as monitor lizards [45], alligators [46], and snakes [47] have relatively high blood pressure compared to other reptiles (some are in the range of human blood pressures). We have identified presence of adult nephrogenesis in boa constrictor and the American alligator, which have recorded blood pressures of 60-120/40-100 mmHg and 75/60 mmHg, respectively [46,47]. Based upon this data, blood pressure and glomerular filtration rate may not be the driving force determining nephrogenesis versus nephron hypertrophy as the mechanism underlying kidney growth.

It is also possible that continual nephron endowment is a strategy to match kidney’s total functional capacity with body growth. Many species of fish and reptile continue to grow during their life span, while growth rate declines during adolescence in mammals [48]. Despite the differences in growth strategies between fish, reptiles, and mammals, the overall ratio of growth from hatching/birth to adulthood is not significantly different [49]. This suggests that the continuous body growth and the ratio of adult-to-newborn body size are not sufficient to explain the switch between nephrogenesis and nephron hypertrophy. Thus, the mechanisms determining continual nephron endowment versus kidney hypertrophy remain to be determined.

### Continual nephrogenesis and branching nephrogenesis

**Fig 7:**
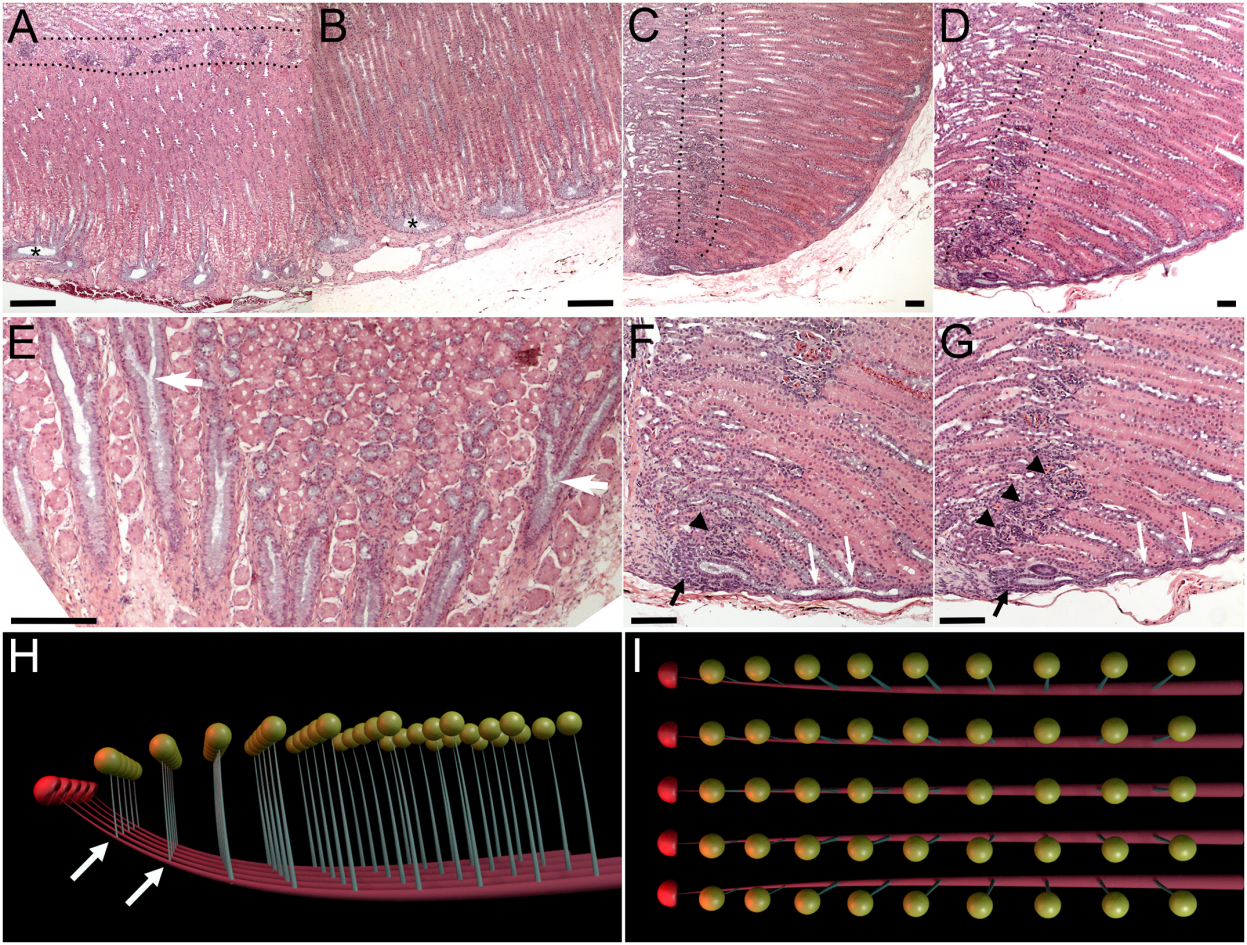
Alligator kidney topology suggests tight correlation between nephrogenesis and branching of the collective system. (A-B) cross-section of near parallel collecting ducts that transverse the kidney in the sub-capsular location (asterisks), giving off near-perpendicular terminal branches that drain the nephrons (glomeruli can be seen in (A) inside the dotted area. (C-D) Longitudinal section through individual collecting ducts near their tips, where new nephrogenesis can be seen (further detailed in F-G). (E) Section taken parallel to and just under the capsule shows near-parallel row of collecting ducts that occasionally split along their course (white arrows). However, most branching events result in generation of terminal duct branches that are seen in cross-section. (F-G) Higher magnification longitudinal sections through individual collecting ducts near the tips. Black arrows point to zones of nephrogenesis. Arrowheads label glomeruli. White arrows show splitting of terminal collecting branches. (H-I) 3D model of Alligator kidney topology at a single lobe level (only the bottom half of the lobe is shown: it is mirrored on top). Each duct (red) gives off sequential terminal branches (grey), each draining a nephron (represented by yellow spheres). Examples of past branching events are marked with white arrows. At the tips of collecting ducts new nephrons are formed (red caps). Scale bars in (A-G) = 100 μm.

In developing mammalian kidney embryonic nephrogenesis is tightly linked to UB branching (thus the term branching nephrogenesis). It would be interesting to see if adult nephrogenesis in reptiles is similarly linked to collecting duct branching. We took advantage of the highly organized nature of the alligator kidney to try and address this question (Fig 7). It appears that branching of the collecting ducts occurs continually and sequentially with the newest branches located near the zones of nephrogenesis (Fig 7C, 7F, 7D and 7G). This is further illustrated in our 3D model of the alligator kidney anatomy (Fig 7H and 7I). Thus our results suggest that alligator kidney continues to undergo collecting duct branching as is adds new nephrons. We currently do not know whether this observation applies to other reptiles, however from considerations of symmetry, we would predict this is likely to be the case.

### Persistence of metanephric progenitor cell niche

We surveyed presence of continual nephrogenesis in juvenile and adult reptiles by detecting presence of condensed mesenchyme on the periphery of kidneys just under the renal capsule (condensing cap mesenchyme), surrounding the tip of ureteric duct branch, which recapitulates nephron development during embryogenesis (Figs 1 and 2). Using the alligator as a model, we were able to detect Six2 expression in the cap mesenchyme in both juvenile and adult animals (Fig 6). The localization of Six2 was very similar in adult alligators and embryonic mouse kidneys. The Six2 transcription factor is critical for maintenance of the nephron progenitor cell population and in part controls appropriate differentiation as cells exit the progenitor cell pool [40,41,50]. The presence of Six2 expressing cells in the alligator suggests that reptiles are able to create nephrons de novo throughout life due to the persistence of the nephrogenic progenitor cells. Unlike other vertebrates, mammals lose the ability for continual nephrogenesis due to the loss of the Six2 expressing progenitor cells [15]. In order to maintain nephrogenesis, there must be a balance between self-renewal and differentiation of progenitor cells. The precise mechanism for maintaining Six2 positive mesenchymal cells in the kidney remains unknown, although it appears to involve signaling between compartments of the metanephric mesenchyme, adjacent ureteric bud epithelium, and renal capsule [51–55]. The presence of Six2 expressing mesenchyme in the nephrogenic zones in juvenile and adult alligator kidneys suggests that the embryonic niche, or at least a modified niche, is stabilized in these animals. Alternatively, Six2-expressing cell population may arise de novo from yet another progenitor cell source before each nephrogenesis event. *A*. *carolinensis* may present a unique model to examine these processes. We were not able to detect evidence of adult nephrogenesis or increased nephron number in Gekkota and in *A*. *carolinensis*. Our sample of Gekkota was limited and may require further confirmation, but in adult *A*. *carolinensis*, we did not detect nephrogenesis despite exhaustive sampling and estimating nephron numbers. Interestingly, green anole is phylogenetically positioned among species demonstrating juvenile and adult nephrogenesis (Fig 3; [35,37]). In our samples of *A*.*carolinensis* we were not able to detect presence of Six2 positive cells. This result correlates with the absence of nephrogenesis in adult green anole. However, this observation needs to be further confirmed by making comparison to a juvenile or embryonic stage that still shows evidence of nephrogenesis (we do not currently know at what point nephrogenesis ceases in *A*. *carolinensis*). We predict that disappearance of nephrogenesis will correlate with disappearance of Six2+ cell population.

On the other extreme, Testudines demonstrate unusually high rates of nephrogenesis compared to other examined reptiles. Mechanisms underlying these high rates of nephrogenesis are currently unknown. Another puzzling observation is the difference in distribution of nephrogenic zones. In particular, alligators show very orderly addition of new nephrons along “lines of nephrogenesis” (S5 Fig). This arrangement sharply contrasted with random distribution of nephrogenic zones in other examined reptiles. This orderly nature of alligator nephrogenesis leads to highly organized architecture of alligator kidney, where all the glomeruli are aligned within two parallel planes within each lobe (Figs 1, 2 and 7). This contrasts sharply with the random arrangement of glomeruli in other examined reptiles, for example turtles (Fig 1H, S1B and S1C Fig). The question remains: what is the evolutionary and ecological significance to this highly organized nature of alligator nephrogenesis, and the resultant kidney anatomy.

Thus, a comparative study of kidney development and continual nephrogenesis in reptiles may provide unique insights into tissue, cellular and molecular mechanisms required to generate and maintain nephrogenic progenitor cell pools. Such study may provide crucial insights to understanding a developmental switch between nephrogenesis and nephron hypertrophy, and development of engineering tools for kidney regeneration.

## Materials and Methods

### Specimen collection and histology

The post-mortem frozen specimens of *C*. *picta* (n = 1), *G*. *gecko* (n = 1), *L*. *burtonis* (n = 1), *C*. *tropidosternum* (n = 1), *T*. *teguxin* (n = 1), *V*. *albigularis* (n = 1), *A*. *carolinensis* (n = 1), *U*. *aegyptia* (n = 1), *P*. *cocincinus* (n = 1), *B*. *constrictor* (n = 1), were a generous gift by Jungle Bob's Reptile World, NY. All specimens obtained from Jungle Bob's Reptile World were pets that died of natural causes. Robert Smith, owner). As an alternative to incinerating them, Mr. Smith donated them to one of the authors (J.L. Conrad) for use in scientific studies. These pet trade specimens were used with permission.

A juvenile specimen of *P*. *obsoletus* (n = 1) was found as a road-kill. Formalin fixed adult specimens of *T*. *scripta* (n = 2) were purchased from (Ward’s Science, Rochester, NY). Additional formalin fixed specimens of *A*. *carolinensis* (n = 10) were purchased from (Nasco, WI). Adult, juvenile and embryonic specimens of *A*. *mississippiensis* (n = 5) kidney were obtained as salvage specimens from alligators collected on Rockefeller Wildlife Refuge (Grand Chenier, LA) for other research projects in collaboration with staff biologists; embryos were preserved as part of an educational dissection exercise for high school students. The alligator kidneys were collected post-mortem by Dr. Ruth Elsey from three juvenile and two adult alligators as well as three archived embryos. The state agency is not university based, and does not have an IACUC. Scientific collecting permits were obtained from Louisiana Department of Wildlife and Fisheries (AV060315) and the collection was performed by employees of the Louisiana Department of Wildlife and Fisheries under supervision of Dr. Ruth Elsey in accordance with the agency’s Best Management Practices guidelines for use and care of alligators. Kidney samples of *T*. *aculeatus* (n = 2) and *O*. *anatinus* (n = 2) were obtained by sampling catalogued specimens at the American Museum of Natural History after obtaining a destructive sampling permit from the AMNH Department of Mammalogy. The specimens used for the study were: M-202818 and M-201412—Tachyglossus aculeatus; M-65819 and M-202081 -Ornithorhynchus anatinus. We did not house or sacrifice any live animals for the purpose of this study.

Most kidneys were submitted entirely. *B*. *constrictor* and *A*. *mississippiensis* kidneys were sampled representatively, including proximal, mid and distal portions. *T*. *aculeatus* and *O*. *anatinus* kidneys were sampled representatively (about half of one kidney from each specimen).

Kidneys were dissected, fixed in formalin and processed for histology using standard techniques. Four-micron sections were stained with Hematoxylin and Eosin and examined using Olympus BX51 microscope.

### Immunofluorescence and confocal microscopy

Tissue from freshly dissected kidneys was fixed in 4% formaldehyde overnight at 4°C, followed by washes in PBS. Tissue was then placed in a 30% sucrose/PBS solution at 4°C until the sucrose solution fully penetrated the tissue. The kidney tissue was then mounted in OCT for cryosectioning. 8μm sections were placed on slides for immunofluorescent staining. Antibody staining and immunofluorescent detection was performed as described [56]. Rabbit polyclonal anti-human Six2 antibody was obtained from Proteintech (11562-1-AP) and was detected using Alexa488 anti-rabbit secondary antibody (Invitrogen). In addition, sections were also stained with Alexa555 wheat germ agglutinin (Invitrogen) and DAPI (Roche). Images were captured using a Nikon C2 confocal microscope and processed in Adobe Photoshop.

### Measurements and statistical analysis

Nephrogenesis counts were obtained manually in those specimens where accurate counts could be obtained (freeze artifact prevented obtaining nephrogenesis counts on all specimens), and normalized to the effective surface area of the kidney. To determine an effective surface area, we scanned each slide on which counts were obtained (using Epson Expression 1680 scanner), and measured surface perimeter using ImageJ. The resultant effective surface area was determined as S = (D+h)*P, where D = average diameter of the zones of nephrogenesis, h = section thickness, P = kidney section perimeter. The surface nephron density was determined as a ratio of the number of nephrogenesis events per section divided by the effective surface area of the kidney section.

To estimate the total relative glomerular number, we performed total glomerular counts per section and divided by an effective section volume defined as V = (D+h)*S, where D = average glomerular diameter, h = section thickness, S = kidney section area (measured on scanned image using ImageJ). The resultant relative total glomerular number was estimated as N = n*W/d*V, where n = glomerular number in the section, W = combined weight of the kidneys, V = effective volume of the section, d = kidney density. We could not assess value of ‘d’ and assumed it to be 1g/ml. Thus, the total estimated glomerular numbers are likely slightly overestimated, but this should not impact the relative number estimates between kidneys of different size in the same species. Glomerular surface area was estimated as D^2, where D is a glomerular diameter. The actual surface area is virtually impossible to exactly measure, but it should remain proportionate to the square of the glomerular diameter, thus we can use D^2 as a surrogate measure of the glomerular surface area. All graphs were plotted in Excel, which was also used to calculate correlation coefficients and estimate slope line parameters.

## Supporting Information

S1 Fig Lower power images demonstrating zones of nephrogenesis (corresponding to Fig 1). (A) *A*. *mississippiensis* (Fig 1*A*), (B) *T*. *scripta* (Fig 1*B*), (C) *C*. *picta* (Fig 1*C*), (D) *T*. *teguxin* (Fig 1*D*), (E) *U*. *aegyptia* (Fig 1*E*), (F) *B*. *constrictor* (Fig 1*F*). Arrows point to zones of nephrogenesis. Scale bar = 100 μm.
(TIF)

S2 Fig Lower power images demonstrating new nephron formation in *A*. *mississippiensis*. (A) corresponds to higher power images in Fig 2E and 2G, (B) corresponds to Fig 2F. Scale bars = 100 μm.
(TIF)

S3 Fig Monotreme adult kidney histology. (A-B) Adult kidney sections from *Tachyglossus aculeatus* (short-beaked echidna) obtained from two individual specimens stained with H&E. (C-D) Sections of adult kidney tissue isolated from *Ornithorhynchus anatinus* (platypus). In all images, the outer cortex and renal capsule are shown. No evidence of nephrogenesis was detected in these species of monotremes, a trait shared with other mammals such as rodents and humans. Scale bar = 50μm.
(TIF)

S4 Fig Examples of kidney histology in specimens that did not show evidence of adult nephrogenesis. (A) *G*. *gecko*, (B) *A*. *carolinensis*, (C) *L*. *burtonis*. The specimens of gekkota were limited. Therefore, we believe the negative results are inconclusive. In contrast, of eleven examined specimens of *A*. *carolinensis*, none showed evidence of nephrogenesis by histology (B, also Fig 4F, left panel), or by estimating total glomerular number (Fig 4). Scale bar = 100 μm.
(TIF)

S5 Fig Gross morphology of nephrogenic zones along renal lobes of the adult American alligator. Nephrogenic zones appear as opaque lines running along the periphery of each renal lobe (arrows). Inset: magnification of nephrogenic zones highlight by dotted lines.
(TIF)

S6 Fig Six2 in *Alligator mississippiensis*. **(A)** Amino acid alignment of Six2 proteins from human and American alligator (XM_006272170). Red color indicates identical residues, dashes represent amino acid stretches present in one but not the other species, and blue indicates non-conserved residues. Six2 proteins from human and American alligator share over 90% amino acid identity. (B) RT-PCR of *Six2* (XM_006272170) from adult American alligator kidney along with *GAPDH* (XM_006258364) loading control. To confirm correct amplicon for Six2, the fragment was gel purified and sequenced.
(TIF)

S7 Fig Lack of Six2 antibody staining in adult kidneys of *Anolis carolinensis*. (XM_003225172). (A) Negative Six2 antibody staining (compare to Fig 6*A* and 6*J*). (B) Wheat germ agglutinin (WGA) staining (used here as a non-specific fluorescent counter-stain). (C) The result of merging (A) and (B). Scale bar = 50 μm.
(TIF)

S1 Table Species collected for analysis with corresponding body mass/length. (DOC)
