# Correction: Postembryonic Nephrogenesis and Persistence of Six2-Expressing Nephron Progenitor Cells in the Reptilian Kidney

**DOI:** 10.1371/journal.pone.0156475

**Published:** 2016-06-01

**Authors:** The PLOS ONE Staff

There is an error in Fig 3. The publisher apologizes for the error. Please see the correct Fig 3 here.

**Fig 3:**
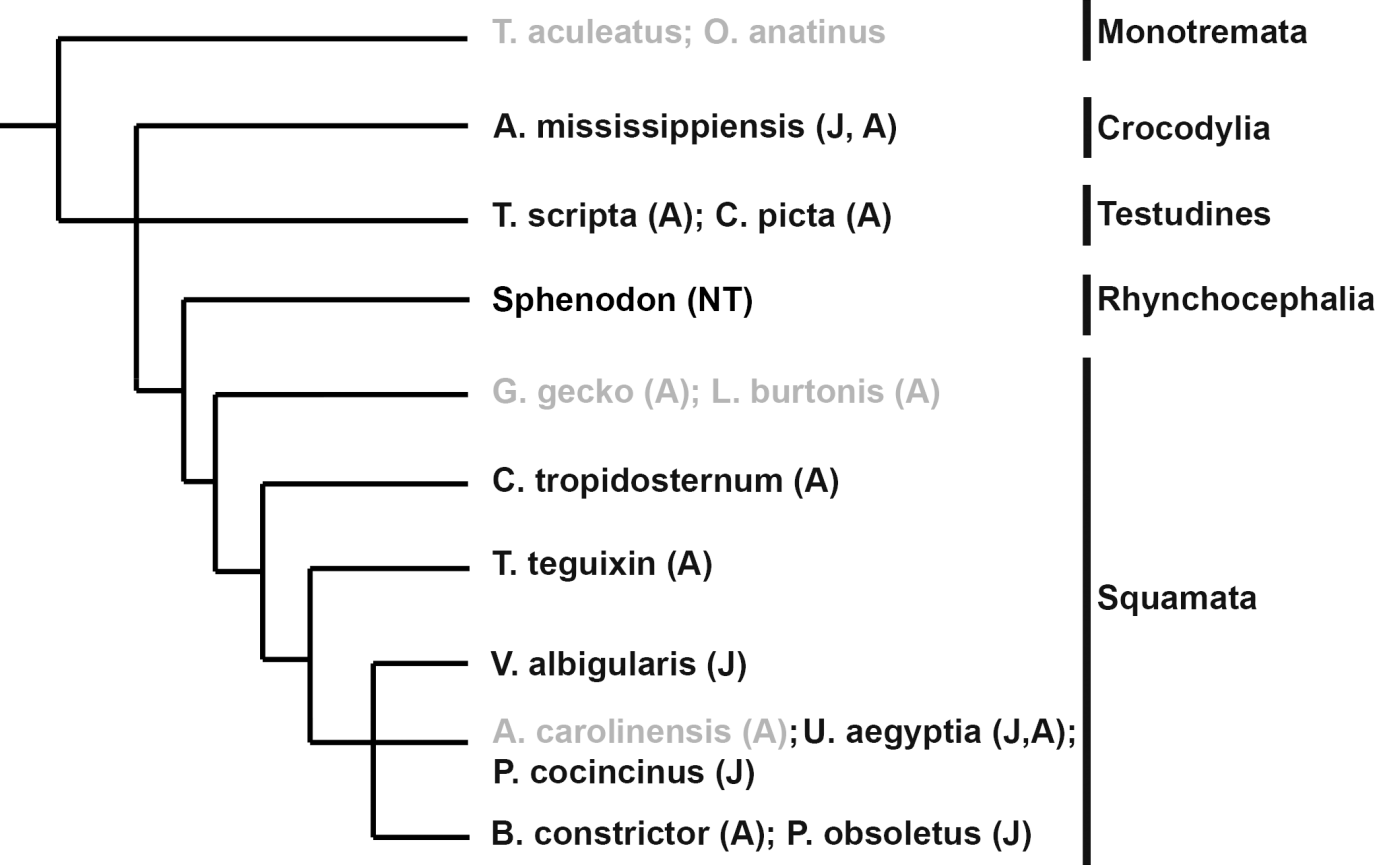
Species surveyed for post-embryonic nephrogenesis. Cladogram displaying species tested for presence of nephrogenesis in juvenile (J) or adult (A) kidney. Two species of monotremes, *Tachyglossus aculeatus* (short-beaked echidna) and *Ornithorhynchus anatinus* (platypus), were used for comparison as the most basal mammalian group. As with other mammals, no evidence of nephrogenesis was detected in the monotreme species. Post-embryonic nephrogenesis was detected in all major reptilian groups surveyed. Species names in light gray did not display evidence of nephrogenesis. NT = not tested.
