# Supplementary figures and images for: Postembryonic Nephrogenesis and Persistence of Six2-Expressing Nephron Progenitor Cells in the Reptilian Kidney
Source: PLoS One. 2016 May 4;11(5):e0153422. doi: 10.1371/journal.pone.0153422 (PMC4856328; doi:10.1371/journal.pone.0153422)

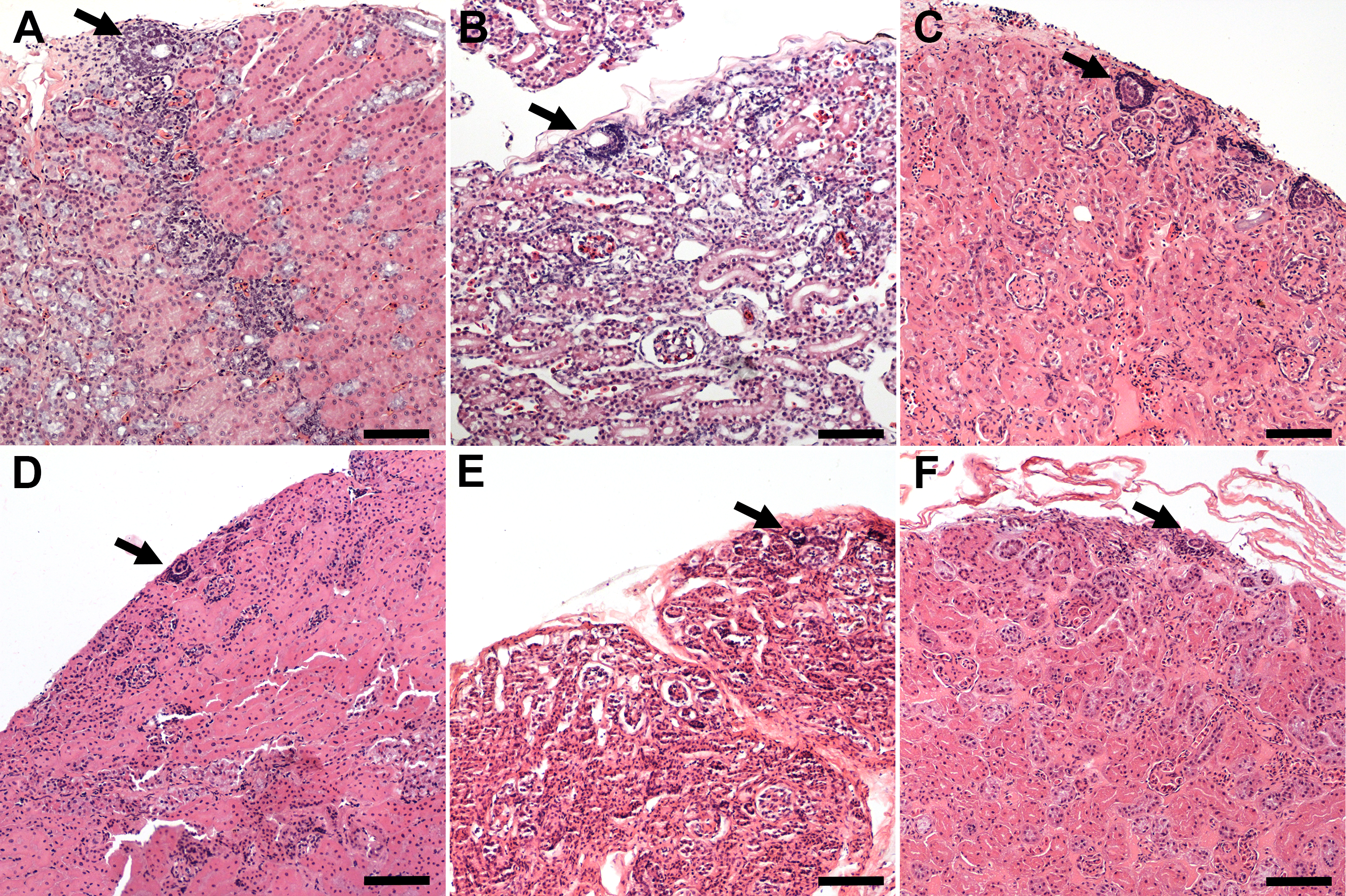

Supplement: S1 Fig — (A) A. mississippiensis (Fig 1A), (B) T. scripta (Fig 1B), (C) C. picta (Fig 1C), (D) T. teguxin (Fig 1D), (E) U. aegyptia (Fig 1E), (F) B. constrictor (Fig 1F). Arrows point to zones of nephrogenesis. Scale bar = 100 μm. (TIF) [file pone.0153422.s001.tif]

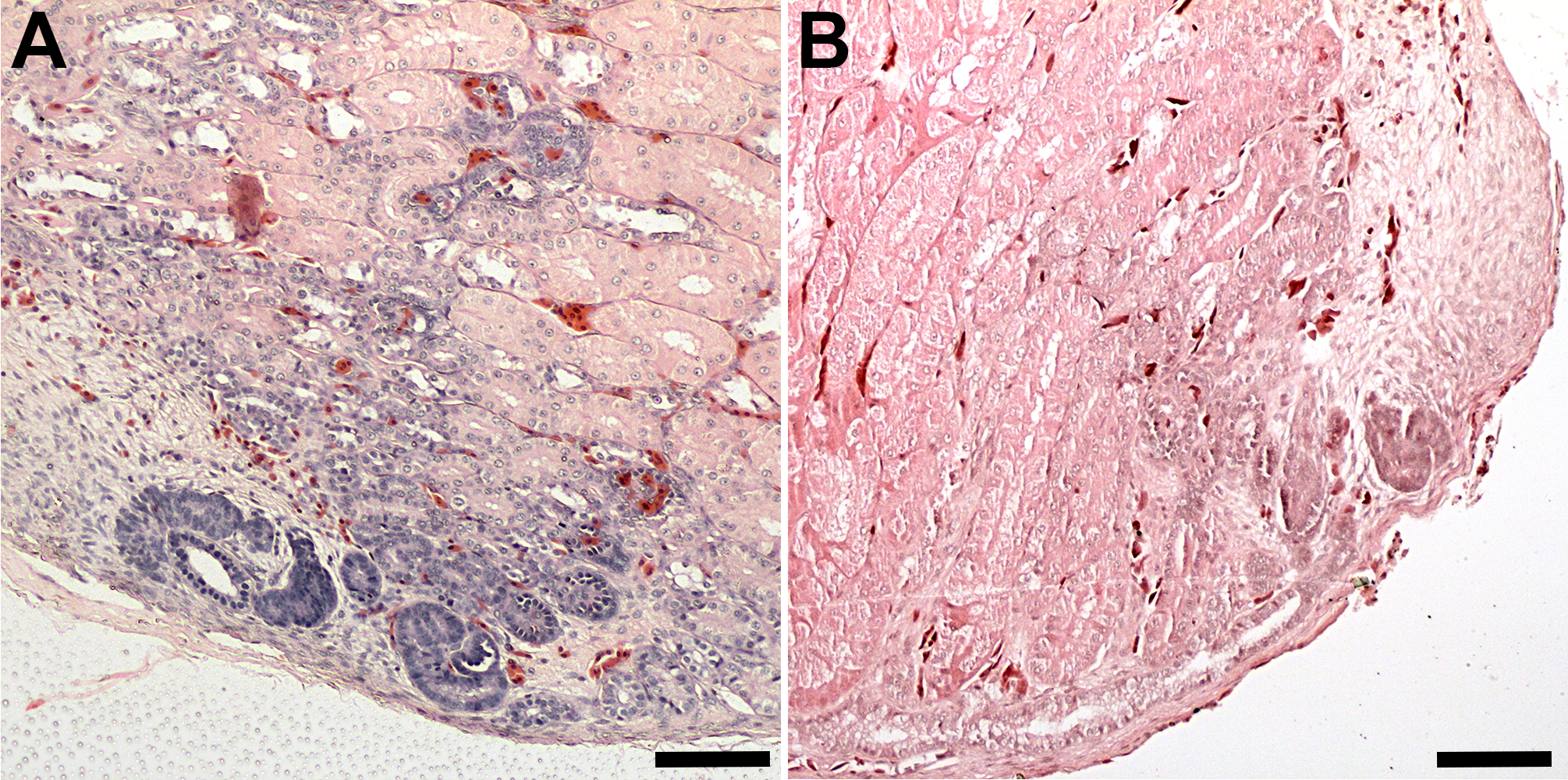

Supplement: S2 Fig — (A) corresponds to higher power images in Fig 2E and 2G, (B) corresponds to Fig 2F. Scale bars = 100 μm. (TIF) [file pone.0153422.s002.tif]

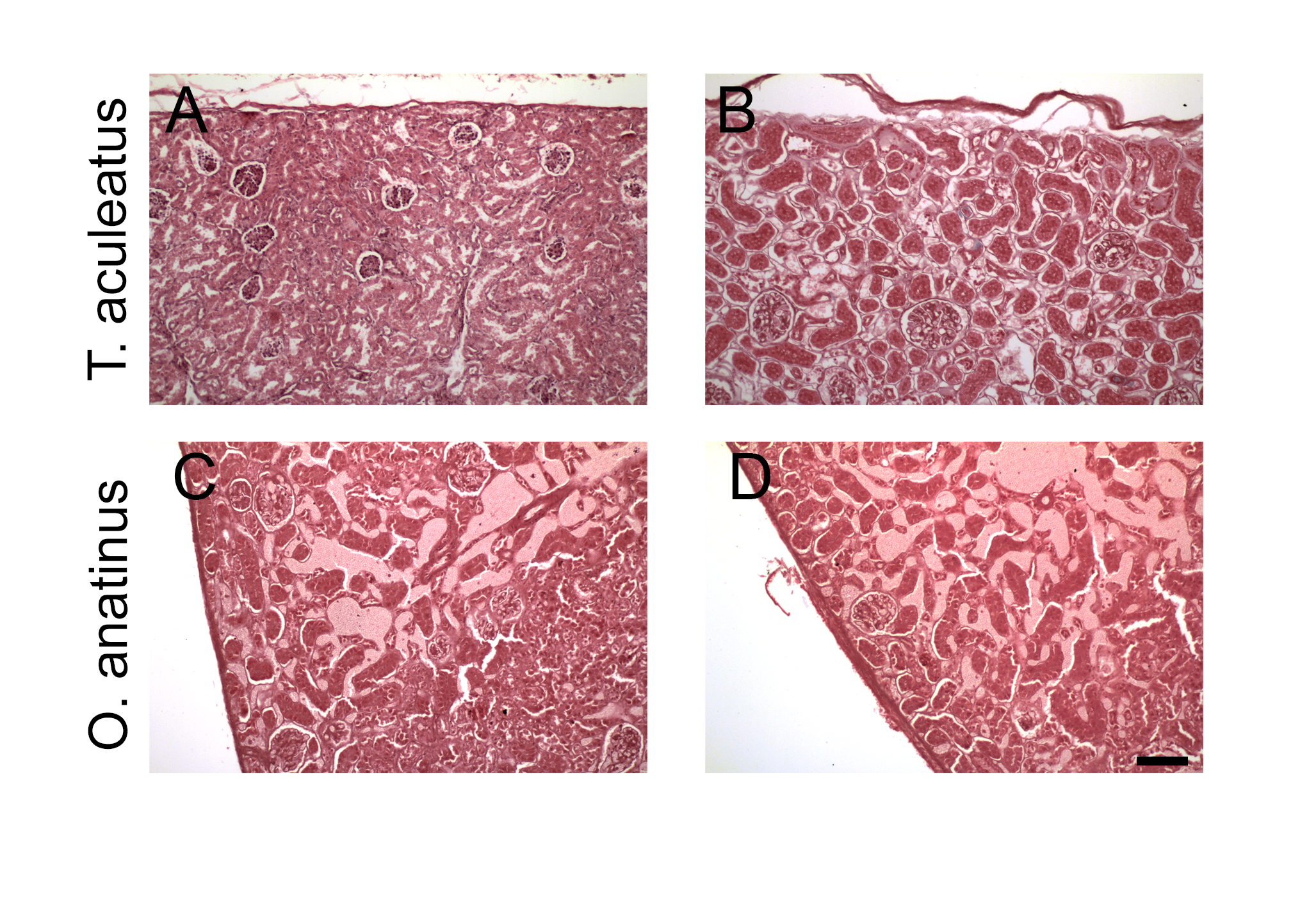

Supplement: S3 Fig — (A-B) Adult kidney sections from Tachyglossus aculeatus (short-beaked echidna) obtained from two individual specimens stained with H&E. (C-D) Sections of adult kidney tissue isolated from Ornithorhynchus anatinus (platypus). In all images, the outer cortex and renal capsule are shown. No evidence of nephrogenesis was detected in these species of monotremes, a trait shared with other mammals such as rodents and humans. Scale bar = 50μm. (TIF) [file pone.0153422.s003.tif]

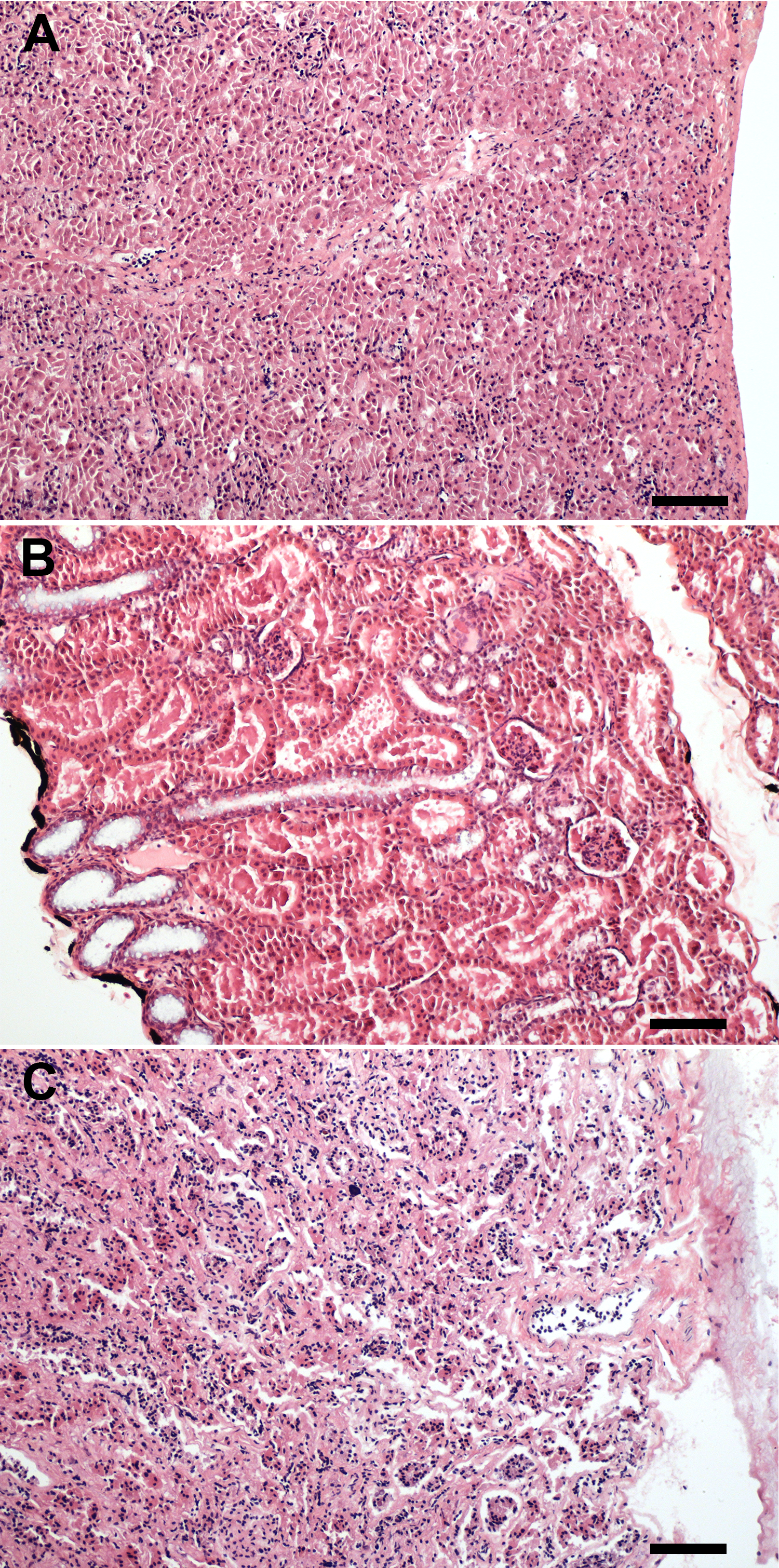

Supplement: S4 Fig — (A) G. gecko, (B) A. carolinensis, (C) L. burtonis. The specimens of gekkota were limited. Therefore, we believe the negative results are inconclusive. In contrast, of eleven examined specimens of A. carolinensis, none showed evidence of nephrogenesis by histology (B, also Fig 4F, left panel), or by estimating total glomerular number (Fig 4). Scale bar = 100 μm. (TIF) [file pone.0153422.s004.tif]

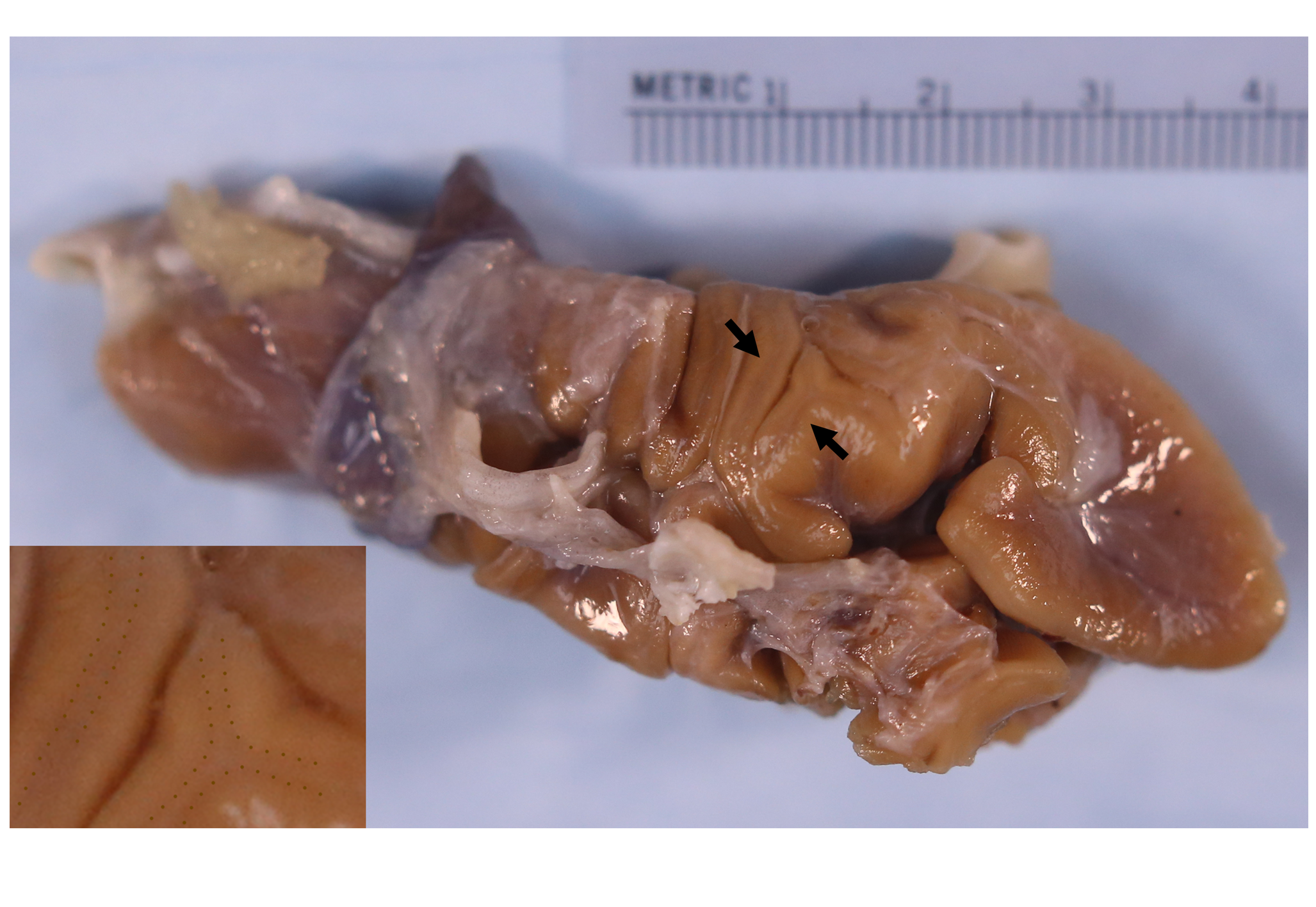

Supplement: S5 Fig — Nephrogenic zones appear as opaque lines running along the periphery of each renal lobe (arrows). Inset: magnification of nephrogenic zones highlight by dotted lines. (TIF) [file pone.0153422.s005.tif]

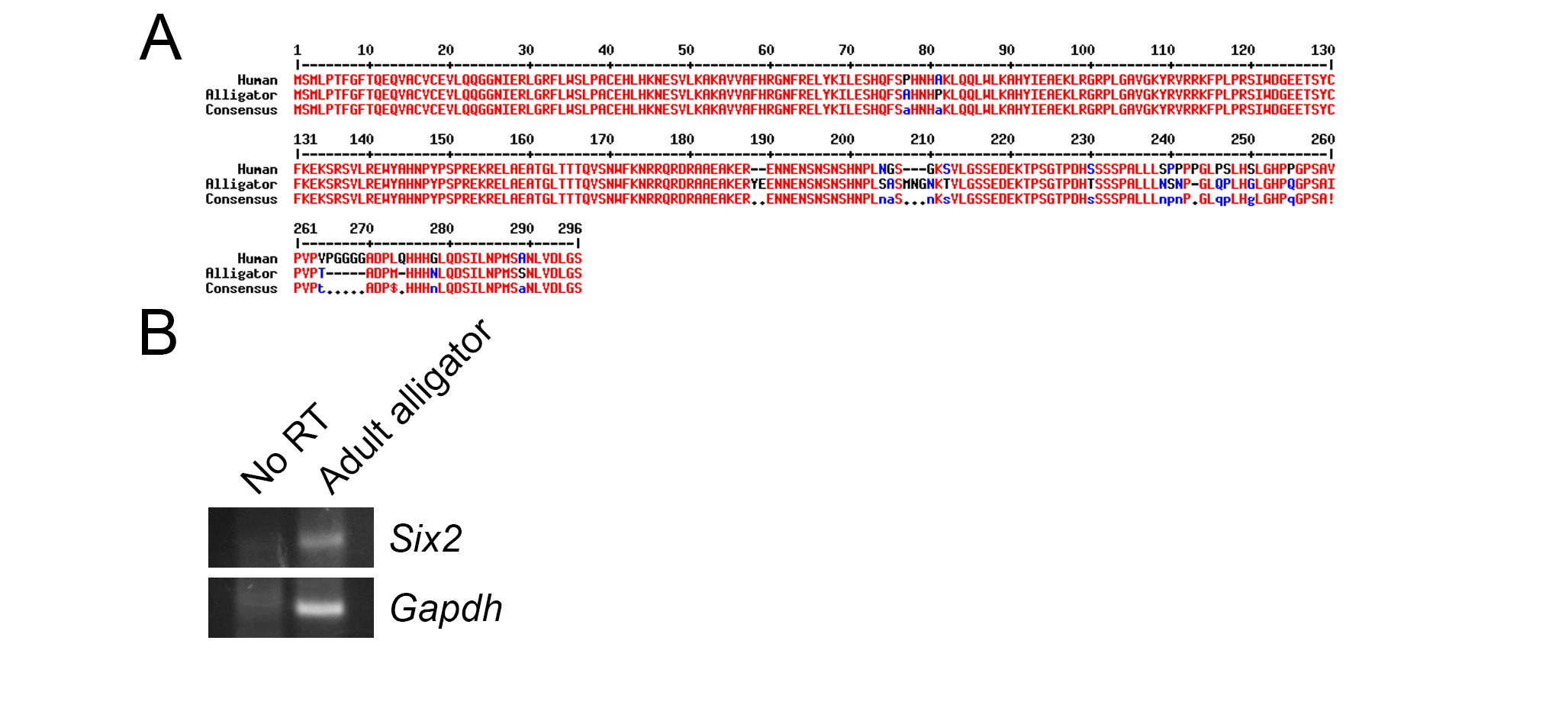

Supplement: S6 Fig — (A) Amino acid alignment of Six2 proteins from human and American alligator (XM_006272170). Red color indicates identical residues, dashes represent amino acid stretches present in one but not the other species, and blue indicates non-conserved residues. Six2 proteins from human and American alligator share over 90% amino acid identity. (B) RT-PCR of Six2 (XM_006272170) from adult American alligator kidney along with GAPDH (XM_006258364) loading control. To confirm correct amplicon for Six2, the fragment was gel purified and sequenced. (TIF) [file pone.0153422.s006.tif]

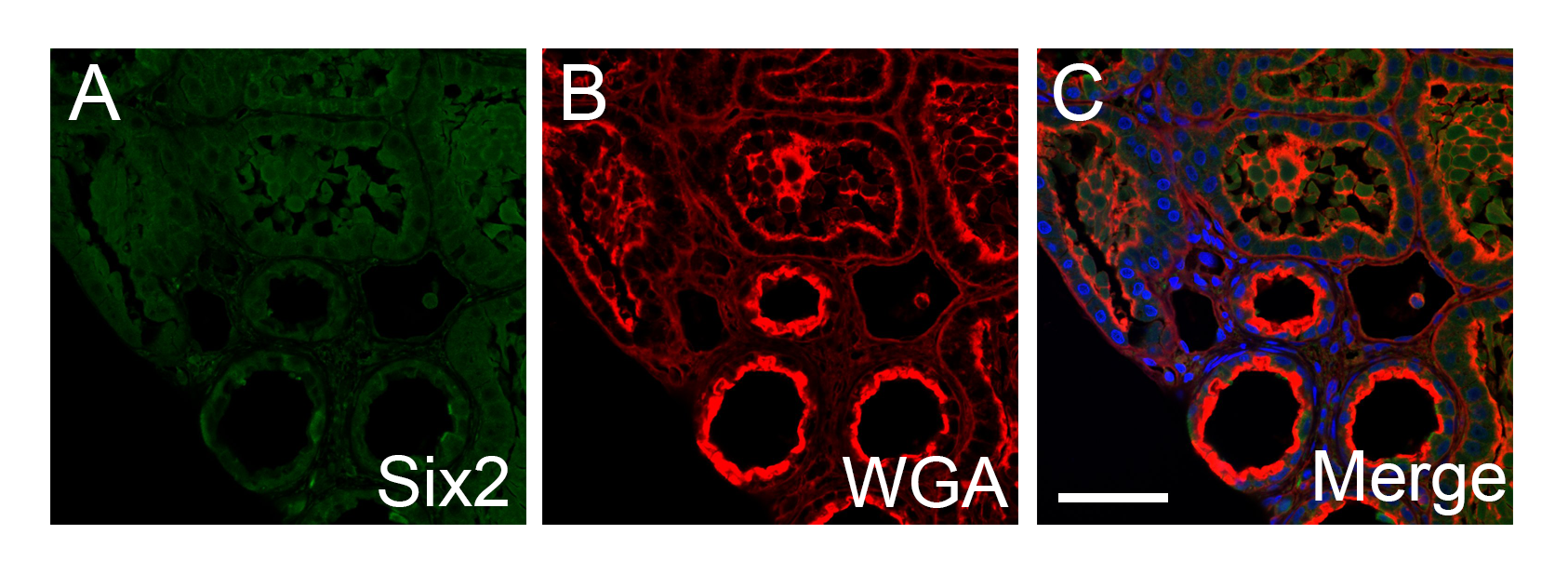

Supplement: S7 Fig — (A) Negative Six2 antibody staining (compare to Fig 6A and 6J). (B) Wheat germ agglutinin (WGA) staining (used here as a non-specific fluorescent counter-stain). (C) The result of merging (A) and (B). Scale bar = 50 μm. (TIF) [file pone.0153422.s007.tif]
