# Supplementary material for: Postembryonic Nephrogenesis and Persistence of Six2-Expressing Nephron Progenitor Cells in the Reptilian Kidney
Source: PLoS One. 2016 May 4;11(5):e0153422. doi: 10.1371/journal.pone.0153422 (PMC4856328; doi:10.1371/journal.pone.0153422)
Supplement: S1 Table — (DOC) [file pone.0153422.s008.doc]

**Table S1: Species collected for analysis**

| **Species** | **Body mass** | **Body Length (cm)** | **Kidney mass** |
| --- | --- | --- | --- |
| *Trachemys scripta elegans* | NA | 17.1 | right= 0.629 grams, left= 0.751 grams |
| *Trachemys scripta elegans* | NA | 22.9 | right= 3.094 grams, left= 3.48 grams |
| *Chrysemys picta* | 257 grams | 14.6 | right= 1.215, left= 0.617 grams |
| *Pantherophis obsoletus* | 17.4 grams | 38.7 | 0.16 grams each |
| *Tupinambis teguixin* | 26.6 grams | 31.8 | 0.06 grams each |
| *Tupinambis teguixin* | 0.206 kg | 44.5 | right= 0.299 grams, left= 0.338 grams |
| *Lialis burtonis* | 18.9 grams | 14.3 | 0.01 grams combined |
| *Varanus albigularis* | 6.19 grams | 16.5 | NA |
| *Cordylus tropidosternum* | 12.6 grams (head missing) | 14.3 | right= 0.027 grams, left (fragments)=0.017 grams |
| *Boa constrictor* | 4.81 kg | 185 | right= 13.10 grams, left= 13.86 grams |
| *Physignathus cocincinus* | 14.1 grams | 26.7 | right= NA, left= 0.008 grams |
| *Uromastyx aegyptia* | 51.6 grams | 30.5 | 0.018 grams (one kidney) |
| *Gekko gecko* | 29.9 grams | 14.3 | right (fragment)= 0.015 grams, left (fragment)= 0.020 grams |
| *Anolis carolinensis* | 3.96 grams (tail missing) | 7.9 | 0.006 grams each |
| *Anolis carolinensis* | 2.04 grams | 12.7 | 0.012 grams |
| *Anolis carolinensis* | 2.50 grams | 14 | 0.021 grams |
| *Anolis carolinensis* | 5.09 grams | 17.8 | 0.047 grams |
| *Anolis carolinensis* | 2.21 grams | 13.3 | 0.022 grams |
| *Anolis carolinensis* | 2.27 grams | 13.3 | 0.015 grams |
| *Anolis carolinensis* | 4.69 grams | 10.2 | 0.052 grams |
| *Anolis carolinensis* | 4.31 grams | 8.9 | 0.037 grams |
| *Anolis carolinensis* | 2.21 grams | 14 | 0.021 grams |
| *Anolis carolinensis* | 1.99 grams | 12.7 | 0.013 grams |
| *Anolis carolinensis* | 2.72 grams | 9.5 | 0.023 grams |
| *Alligator mississippiensis* | NA | 88.9 | 8.34 grams |
| *Alligator mississippiensis* | NA | 104 | 18.07 grams |
| *Alligator mississippiensis* | NA | 163 | 45.79 grams |
| *Alligator mississippiensis* | NA | 198 | 39.00 grams |
| *Alligator mississippiensis* | NA | 203 | 89.10 grams |
| Tachyglossus aculeatus | NA | 30 | NA |
| Tachyglossus aculeatus | NA | 29 | NA |
| Ornythorrhynchus anatinus | NA | 28 | NA |
